# Fluorescence-based reagent and spectrum-based optical reader for lactoferrin detection in tears: differentiating Sjögren’s syndrome from non-Sjögren’s dry eye syndrome

**DOI:** 10.1038/s41598-024-65487-2

**Published:** 2024-06-24

**Authors:** Chia-Ying Tsai, Chitsung Hong, Min-Yen Hsu, Tso-Ting Lai, Ching-Wen Huang, Cheng-Yo Lu, Wei-Li Chen, Chao-Min Cheng

**Affiliations:** 1 Department of Ophthalmology, Fu Jen Catholic University Hospital, Fu Jen Catholic University, New Taipei City, Taiwan; 2 School of Medicine, College of Medicine, Fu Jen Catholic University, New Taipei City, Taiwan; 3 Graduate Institute of Clinical Medicine, College of Medicine, National Taiwan University, Taipei, Taiwan; 4 SpectroChip Inc., Hsinchu, Taiwan; 5 Department of Ophthalmology, Chung Shan Medical University Hospital, Taichung, Taiwan; 6 School of Medicine, Chung Shan Medical University, Taichung, Taiwan; 7 Department of Ophthalmology, National Taiwan University Hospital, 7 Chung-Shan South Rd, Taipei, 100 Taiwan; 8 Department of Ophthalmology, College of Medicine, National Taiwan University, Taipei, Taiwan; 9 Institute of Biomedical Engineering, National Tsing Hua University, No. 101, Sec. 2, Guangfu Rd., East Dist., Hsinchu, 300 Taiwan

**Keywords:** Biomarkers, Diagnosis, Diseases, Corneal diseases

## Abstract

Identification of an early biomarker and effective testing device to differentiate dry eye disease secondary to autoimmune disease (Sjögren’s syndrome dry eye disease) from non-Sjögren’s dry eye disease are prerequisites for appropriate treatment. We aimed to demonstrate the capacity of a new photo-detection device to evaluate tear lactoferrin levels as a tool for differentiating systemic conditions associated with dry eye disease. Patients with non-Sjögren’s and Sjögren’s syndrome dry eye disease (n = 54 and n = 52, respectively) and controls (n = 11) were enrolled. All participants completed the Ocular Surface Disease Index questionnaire. Tear collection was performed with Schirmer test, and tear break-up time was examined using a slit lamp. Tear lactoferrin was evaluated using our newly developed photo-detection device. The average lactoferrin concentration was significantly lower in samples from patients with non-Sjögren’s dry eye disease (0.337 ± 0.227 mg/mL, n = 54) and Sjögren’s syndrome dry eye disease (0.087 ± 0.010 mg/mL, n = 52) than in control samples (1.272 ± 0.54 mg/mL, n = 11) (p < 0.0001). Further, lactoferrin levels were lower in patients with Sjögren’s syndrome dry eye disease than in those with non-Sjögren’s dry eye disease (p < 0.001). Our cost-effective, antibody-free, highly sensitive photo-detection device for evaluating tear lactoferrin levels can assist ophthalmologists in differentiating different types of dry eye diseases.

## Introduction

Dry eye disease (DED) is a common ocular surface disease that affects millions of people around the world^1^. It is associated with lacrimal gland function, tear osmolality, ocular surface inflammation, and neurosensory abnormalities^2^. In addition to foreign body sensation and soreness in mild cases^3^, large epithelial defects can occur in DED, resulting in pain or even blindness in severe cases^4^. First-line treatment of DED includes artificial tears and warm packing. In severe cases, advanced therapies, such as punctal occlusion, autologous serum^5^, platelet-derived products^6^, intense pulse light therapy^7^, or other meibomian gland treatments^8,9^, may be necessary. Systemic diseases that affect the lacrimal glands, such as primary Sjögren’s syndrome (SS)^10^ and secondary SS caused by autoimmune disease (AI)^11^, can result in severe DED. Patients with these underlying systemic conditions often require systemic treatment to manage their refractory DED. However, it can be challenging to distinguish between DED secondary to AI from non-Sjögren’s DED solely based on symptoms and signs observed in an ophthalmic clinic. Therefore, there is a critical need to not only identify and use a biomarker that can differentiate DED secondary to systemic disease from non-Sjögren’s DED but also to develop an accurate, simple, and cost-effective method to help physicians determine the treatment methodology.

Lactoferrin, also known as lactotransferrin, is a non-heme iron-binding protein^12^. This glycoprotein is secreted by mucosal epithelial cells and is expressed in milk, tears, and saliva^13–16^. Lactoferrin has antiviral and antibacterial properties due to its iron-binding capacity, thus depriving a virus of nutrients^15^, as well as lipid A domains of the bacterial lipopolysaccharide^17^. It also has anti-inflammatory properties owing to its ability to downregulate inflammatory mediators and inhibit the host complement reaction^18^. Tear lactoferrin is secreted by acinar cells within the lacrimal glands^19^ and constitutes 25% of the total weight of tear protein^20^. The normal concentration of lactoferrin in tears is 1.14–2.2 mg/mL^20–22^. Decreased levels of lactoferrin have been reported in tears from patients with ocular surface diseases, such as DED^23–25^, chronic meibomitis, and keratoconus^26^. Tear lactoferrin levels have also been reported to be lower in patients with SS-DED and non-Sjögren’s DED than in healthy individuals^27^. In another small-scale study, patients with graft-versus-host disease (GVHD) presented with lower lactoferrin levels than non-GVHD patients, and tear lactoferrin levels were associated with DED severity^28^. These findings suggest that lactoferrin can play an essential role in differentiating different types of DEDs.

Lactoferrin levels are usually measured using gel electrophoresis^29^, enzyme-linked immunosorbent assay (ELISA)^21^, high-performance liquid chromatography^30^, mass spectrometry^29,31^, lactoplates, or other specialized diagnostic kits^32,33^. However, conventional methods using antibodies are time-consuming, costly, and technique-sensitive. Moreover, the currently available commercial kit (disposable microcassette) for tear lactoferrin level analysis provides only qualitative results.

To substantiate tear lactoferrin level evaluation as a potentially routine clinical biomarker to differentiate patients with DED with and without AI, we collected tear fluid from patients with non-Sjögren’s DED, SS-DED (including primary and secondary SS), and controls, and analyzed lactoferrin levels using a self-designed photo-detection device. The objective of this study was to demonstrate the capacity of our novel photo-detection device to evaluate tear lactoferrin levels as a tool for differentiating systemic conditions associated with DED.

## Materials and methods

### Patient selection

This prospective study was approved by the Institutional Review Board of Fu Jen Catholic University Hospital (No. FJUH110122) and National Taiwan University Hospital (No. 202110062RINB). Written informed consent was obtained from all patients, and the study was performed in accordance with the Declaration of Helsinki. Patients were enrolled from the ophthalmology clinic at Fu Jen Catholic University Hospital and from the National Taiwan University Hospital between August 1, 2021, and June 30, 2022. Patients with non-Sjögren’s DED and SS-DED (including primary and secondary SS) were eligible for the study. Volunteers without ocular symptoms or signs were enrolled as controls. The study excluded patients who underwent ocular surgery in the prior 6 months, or had ocular infections, active ocular inflammatory diseases (other than DED), or high intraocular pressure. None of the patients had used topical ocular medication other than artificial tears or had worn contact lens in the prior 2 weeks.

### Examination of patients and controls

Each patient and normal control underwent a slit-lamp examination for corneal condition evaluation, non-invasive tear break-up time (TBUT) measurement, and the Schirmer test (basic secretion test). All participants completed the Ocular Surface Disease Index (OSDI) questionnaire for subjective evaluation. DED was diagnosed based on the Dry Eye Workshop (DEWS) II criteria; each patient had an OSDI score > 13 and a TBUT < 10 s^34^. All patients with DED were further classified into non-Sjögren’s DED and SS-DED based on their clinical history, laboratory data (including the erythrocyte sedimentation rate [ESR], C-reactive protein [CRP], and antinuclear antibody [ANA] levels, C3, C4, double-stranded DNA, RA factor, as well as anti-Ro and anti-La), and an evaluation by a rheumatologist (for SS-DED). The SS-DED included primary and secondary SS-DED. The classification was primarily based on the clinical history, which includes at least 2 consecutive diagnoses of Sjogren's syndrome and other autoimmune disease, such as Systemic Lupus erythematosus (SLE) and Rheumatoid arthritis (RA) (based on the International Classification of Diseases (ICD); ICD-9: 710.2 or ICD-10: M35.00 for Sjogren’s syndrome, ICD-9: 710.0 or ICD-10: M32.10 for SLE, ICD-9:714.0 or ICD-10:M06.9 for RA) at a rheumatologist’s clinic in one of the participating hospitals to be classified as SS-DED. Other participants without a prior diagnosis of SS, SLE, or RA, but with any complaint of potential symptoms of autoimmune diseases (includes but not limited to joint pain, joint swelling, skin rash, dry mouth, swollen salivary glands, persistent dry cough, or prolonged fatigue) were referred to a rheumatologist’s clinic for further evaluation and blood tests were performed based on the rheumatologists’ judgement. Volunteers were included as controls only if they had a non-invasive TBUT > 10 s, a Schirmer test result > 15 mm, and a complete absence of DED symptoms.

### Tear collection

Tear fluid was collected from the fornix using Schirmer paper strips. Under local anesthesia, Schirmer paper strips were placed in the patients’ fornix for 5 min, the collected tear fluid was resolved in 50 µL of distilled water for 15 min, and samples were stored at − 80 °C for further analysis.

### Evaluation of lactoferrin using a photo-detection device

**Figure 1:**
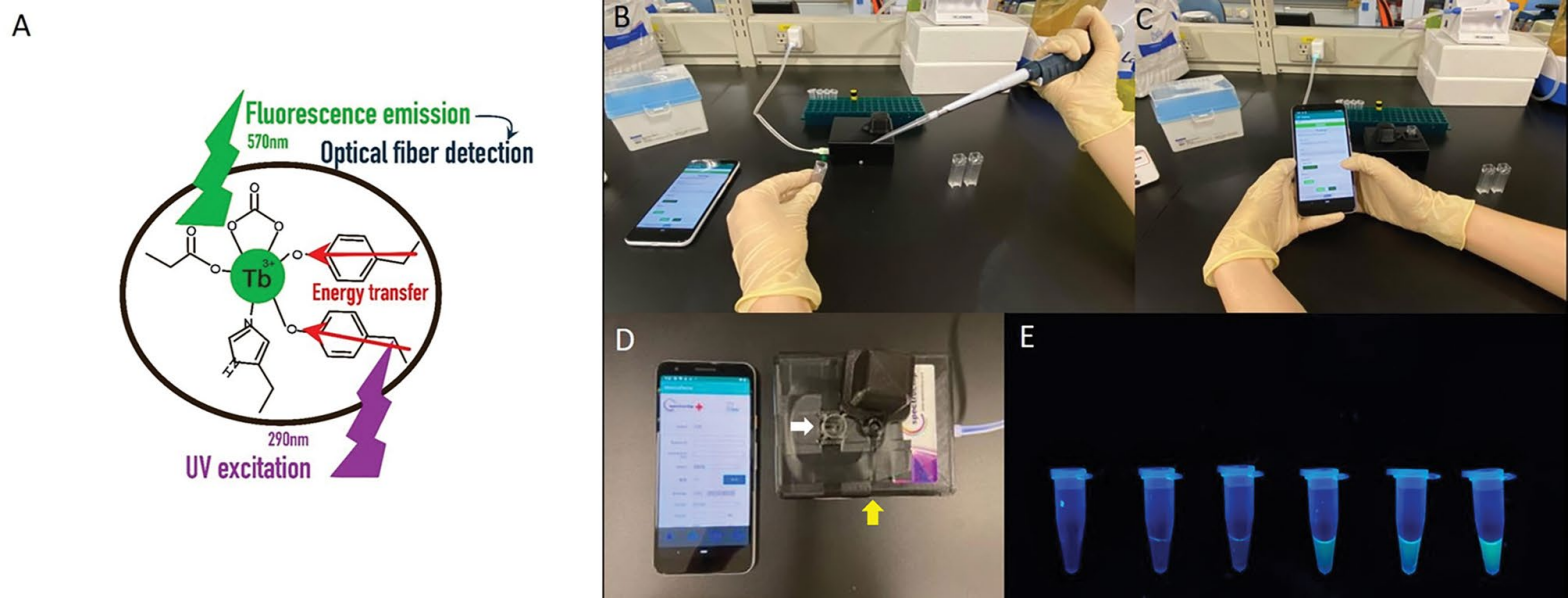
The principle and demonstration of the photo-detection device are shown. (**A**) The Tb^3+^–lactoferrin complex gets excited by ultraviolet light at 290 nm. The energy is transferred through tyrosine at the binding site, and fluorescein-emitted light at a wavelength of 570 nm is obtained, detected by optical fiber and amplified by wafer technology. (**B**) Samples are added to the cuvette. (**C**) Fluorescence levels are checked using the corresponding APP. (**D**) The photo-detection device (yellow arrow), cuvette (white arrow), and its APP are shown. (**E**) Different fluorescence intensities under different concentrations of lactoferrin are shown. *APP* application.

We added 20 µL of diluted tear sample to a cuvette, along with 10 µL (100 µM) of TbCl_3_ solution and 20 µL of distilled water, creating a total volume of 50 µL. The cuvette was then placed in the dark box of the device. On adding TbCl_3_ to the lactoferrin-containing samples, Tb^3+^ bound to the tyrosine kinase metal-binding sites of lactoferrin. The Tb^3+^–lactoferrin complex was excited by ultraviolet light at 290 nm and the resulting energy transferred through the tyrosine at the binding site, causing fluorescein to emit light at a wavelength of 570 nm^28^, which was detected with an optical fiber and amplified using wafer technology (Fig. 1A).

The intensity of the fluorescent light was then used to calculate the sample lactoferrin concentration, based on standard lactoferrin level references, and was recorded using the device’s corresponding app (Fig. 1B–D). Standard lactoferrin level references were established by detecting the fluorescence intensities of different concentrations of diluted lactoferrin from human milk (L0520, Sigma-Aldrich, Darmstadt, Germany) (Fig. 1E).

### ELISA

All standard and tear samples from patients and controls were analyzed using the Human Lactoferrin ELISA Kit (ab200015, Abcam, Danvers, U.S.A.), according to the manufacturer’s instructions.

### Statistical analysis

Data were analyzed using Prism software (ver. 6.04 for Mac; GraphPad Software, Inc., San Diego, CA, USA). One-way analysis of variance (ANOVA) was used to compare the results between non-Sjögren’s DED, SS-DED, and controls. Simple and multivariate linear regressions were used to evaluate the correlation between tear lactoferrin level and other factors including age, Schirmer test result, OSDI, and the etiologies of DED. Sensitivity analysis was performed to exclude the possible confounding effect of systemic immunosuppressant treatment. Statistical significance was set at p < 0.05. The study process flowchart, from patient collection to data analysis, is shown in Supplementary Fig. S1.

## Results

We included 54 patients with non-Sjögren’s DED, 52 with SS-DED, and 11 healthy controls. The average age of the patients was 61 ± 16, 61 ± 12, and 46 ± 23 years in the non-Sjögren’s DED, SS-DED, and control groups, respectively. Female predominance was noted in all subgroups (48 of 54 patients with non-Sjögren’s DED, 49 of 52 patients with SS-DED, and 9 of 11 in the controls), and the percentages were similar among the three subgroups (p = 0.8441). In ophthalmic exams, patients with non-Sjögren’s DED and SS-DED demonstrated significantly lower Schirmer test results than controls (p < 0.0001); however, there was no significant difference between non-Sjögren’s DED and SS-DED (p > 0.05). OSDI scores were significantly higher in patients with non-Sjögren’s DED (p = 0.0295) and SS-DED (p = 0.0001) than in healthy controls. Additionally, patients with SS-DED had higher OSDI scores than patients with non-Sjögren’s DED (p = 0.002) (Table 1).

**Table 1:** Demographics and clinical test results of the study population.

Parameters	Control group	Non- Sjögren’s DED	SS-DED	*p*-value (n = 117)
No. of patients	11	54	52	
Age (yrs)	46 ± 23	61 ± 16	61 ± 12	0.1142 (Con vs non-Sjogren DED) 0.1431 (Con vs SS-DED) 0.9903 (non-Sjogren DED vs SS-DED)
Sex (male/female)	2/9	6/48	3/49	0.8441
OSDI (Score)	7.17 ± 7.63	20.02 ± 9.52	27.59 ± 10.77	0.0295* (Con vs non-Sjögren’s DED); 0.0001* (Con vs SS-DED); 0.002* (non-Sjögren’s DED vs SS-DED);
Schirmer (mm)	14.14 ± 8.84	3.94 ± 2.44	3.65 ± 3.66	< 0.0001* (Con vs non-Sjögren’s DED); < 0.0001* (Con vs SS-DED); 0.6327 (non-Sjögren’s DED vs SS-DED)

Values are presented as number or mean ± standard deviation.

*DED* dry eye disease, *SS-DED* Sjögren’s syndrome DED, *OSDI* Ocular Surface Disease Index, *Con* control.

One-way analysis of variance and Tukey's multiple comparisons test (*p < 0.05).

**Figure 2:**
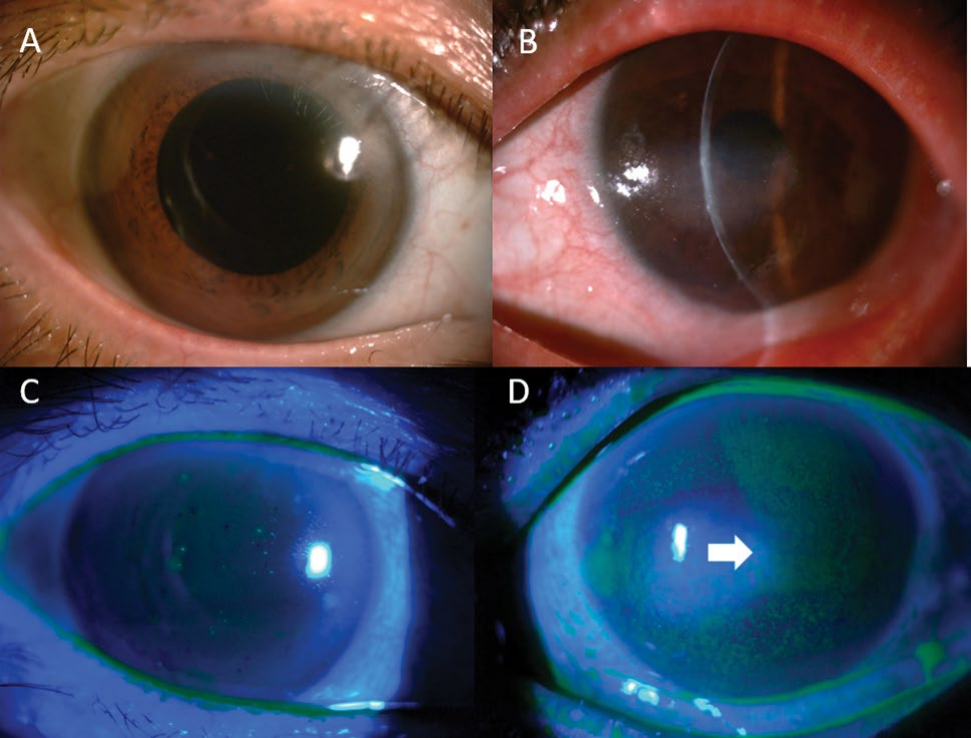
Representative pictures of the outer segment of eyes from patients with non-Sjögren’s DED (**A**,**C**) and SS-DED (**B**,**D**) are shown. (**A**) Picture from a patient with non-Sjögren’s DED, with a Schirmer test result of 4 mm, OSDI of 32, and tear lactoferrin level of 0.49 mg/mL, is shown. (**B**) Picture from a patient with SS-DED, with a Schirmer test result of 1 mm, OSDI of 33, and tear lactoferrin level of 0.05 mg/mL, is shown. (**C**) The fluorescein staining result of the patient in (**A**) is shown, with no obvious superficial punctate keratitis. (**D**) The fluorescein staining picture of the patient in (**B**) is shown, revealing diffuse corneal erosions (arrow). *DED* dry eye disease, *OSDI* Ocular Surface Disease Index, *SS-DED* Sjögren’s syndrome DED.

Among patients with AI, the ESR, CRP, C3, and C4 levels were stable or within normal limits, indicating stable disease conditions. Representative images of the outer segments of patients with non-Sjögren’s DED and SS-DED are shown in Fig. 2. The calibration line of our photo-detection device was developed based on standard samples with different concentrations of lactoferrin (R^2^ = 0.9943) (Supplementary Fig. S2).

**Figure 3:**
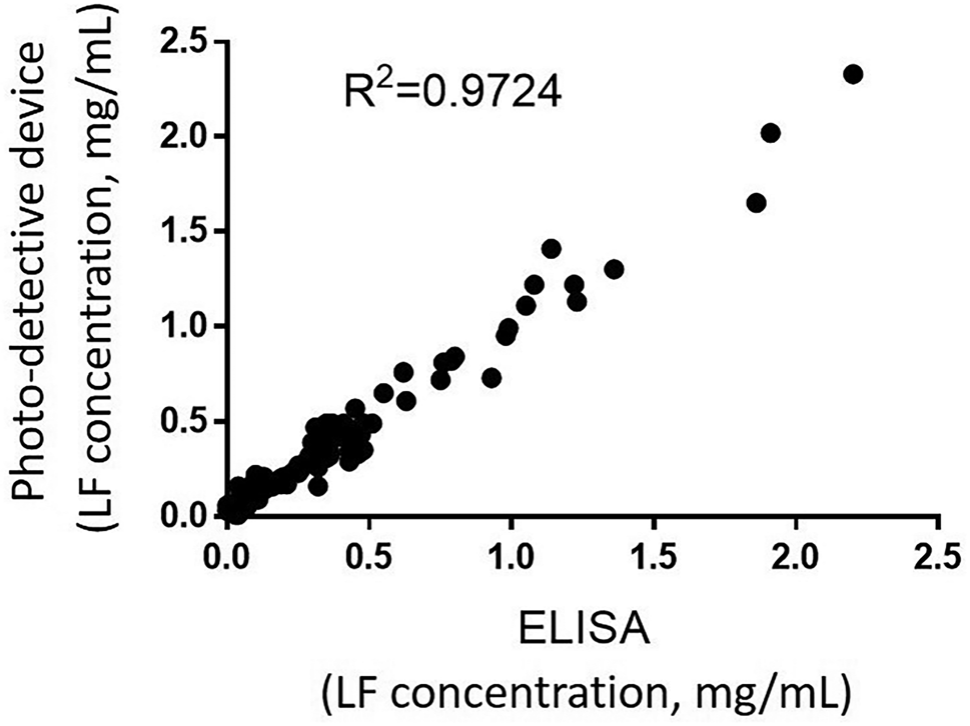
The correlation between the lactoferrin (LF) concentration as assessed using the photo-detection device and with ELISA is depicted. The concentration of LF as assessed using the photo-detection device is highly correlated to that as assessed using ELISA. R^2^ = 0.9724. X-axis: the results from ELISA; Y- axis: the results from the photo-detection device. *ELISA* enzyme-linked immunosorbent assay, *LF* lactoferrin.

In the present study, all standard lactoferrin and tear sample lactoferrin levels were examined using ELISA and our novel photo-detection device. Tear sample lactoferrin levels, as evaluated using ELISA and our device, were highly correlated (R^2^ = 0.9724) (Fig. 3).

**Figure 4:**
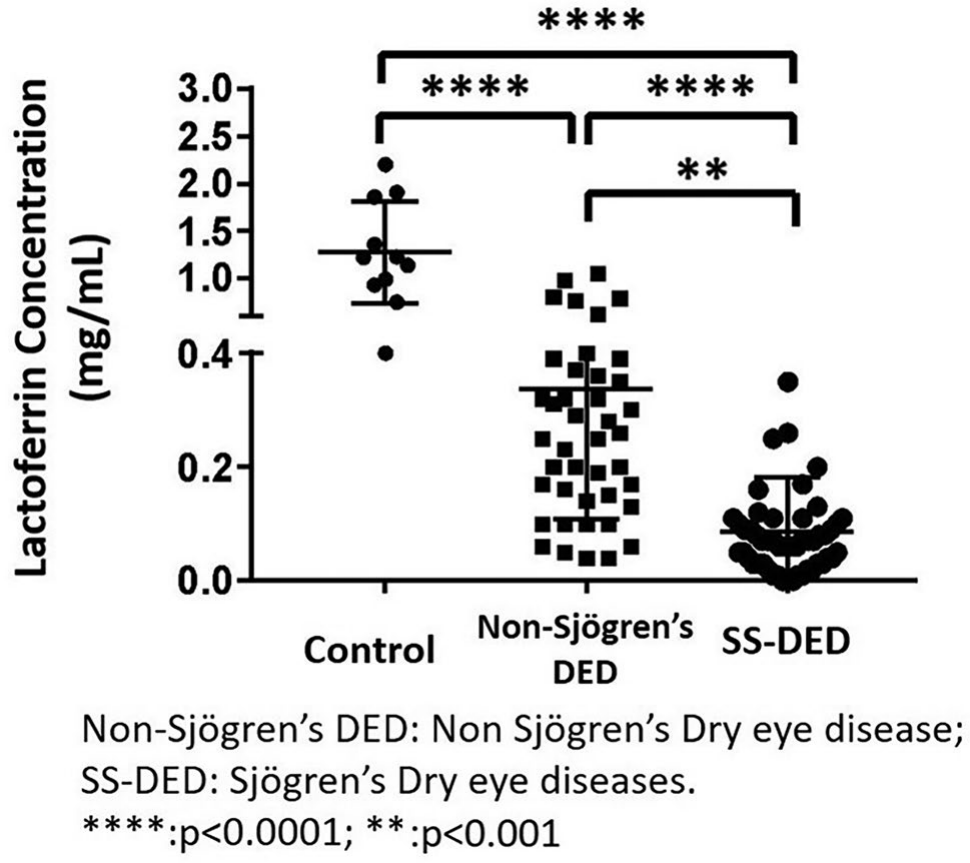
Comparisons in tear lactoferrin levels between non-Sjögren’s DED, SS-DED, and control groups are shown. Patients with non-Sjögren’s DED and SS-DED show significantly lower levels of tear lactoferrin than healthy controls (p < 0.0001). Further, patients with SS-DED show lower tear lactoferrin levels than patients with non-Sjögren’s DED (p < 0.001). *DED* dry eye disease, *SS-DED* Sjögren’s syndrome DED.

The average lactoferrin concentrations were 0.337 ± 0.227, 0.087 ± 0.099, and 1.272 ± 0.54 mg/mL in samples from patients with non-Sjögren’s DED, SS-DED, and controls, respectively. Tear lactoferrin levels were significantly lower in patients with non-Sjögren’s DED and SS-DED than in controls (p < 0.0001). Tear lactoferrin levels were even lower in patients with SS-DED than in patients with non-Sjögren’s DED (p < 0.001) (Fig. 4). During sensitivity analysis, the difference remained significant after excluding the cases under systemic immunosuppressant therapy (p < 0.001).

The regression results between tear lactoferrin levels and other factors are summarized in Table 2. In simple linear regression, tear lactoferrin level was significantly associated with OSDI (beta-coefficient = − 0.359, p < 0.001) and the etiology of DED (i.e. non-Sjögren’s vs SS; beta-coefficient = − 0.582, p < 0.001). Furthermore, in the multivariate linear regression, we found that the tear lactoferrin levels remained significantly associated with the etiology of DED (beta-coefficient = 0.526, p < 0.001) after adjusted for age and the DED severities.

**Table 2:** Association between tear lactoferrin level and other factors.

Variables	Simple linear regression		Multivariate linear regression	
	Beta-coefficient	p-value	Beta-coefficient	p-value
Age	− 0.147	0.133	− 0.136	0.086
Schirmer	0.144	0.140	0.054	0.515
OSDI	− 0.359	< 0.001	− 0.152	0.089
Types of DED	− 0.582	< 0.001	− 0.526	< 0.001

*DED* dry eye disease, *OSDI* Ocular Surface Disease Index.

## Discussion

In the present study, we developed a convenient, rapid, and economical method to detect tear lactoferrin levels for potential use as a biomarker to assist the assessment of DED severity, provide the clue in investigating the autoimmune diseases etiologies and determine the proper treatment. In our study, tear samples were collected using Schirmer strips, a non-invasive, standard examination routinely used in clinics for patients with DED, which waived the need for additional procedures to collect tear samples, minimizing patient discomfort and cost. Lactoferrin levels were analyzed using our photo-detection device, which is antibody-free and inexpensive compared to the gold standard of ELISA for lactoferrin level quantification. The results of the photo-detection device were reliable and in agreement with the ELISA results. Our study findings showed that tear lactoferrin levels were significantly lower in patients with SS-DED than in patients with non-Sjögren’s DED and healthy controls. Further, the study showed that our self-designed photo-detection device was able to accurately and quantitatively measure lactoferrin levels in tears, providing a simple and cost-effective method for clinical use.

In 2019, Sonobe et al.^28^ reported the detection of lactoferrin using a novel microfluidic paper-based analytical device (µPAD) that visualized the fluorescence emitted by the Tb–lactoferrin complex. We also used a photo-detection method; however, our method was improved in several aspects. First, our device was sensitive enough to detect lactoferrin levels as low as 0.1 mg/mL, which is lower than that reported by Sonobe et al.^28^ (approximately 0.2 mg/mL). This was achieved by using a fixed wavelength of emitted fluorescence light to decrease the interference of noise and a signal amplification wafer to increase device sensitivity. This improvement further expands the application of our method to patients with low levels of tear lactoferrin, such as those with SS-DED or GVHD-DED who have scarce amounts of tear fluid and reduced levels of lactoferrin^35^. Second, we employed a cost-effective and less invasive method for tear collection, the Schirmer strip paper. Compared with the use of microcapillary tubules, our method required no additional material for tear collection.

Clinically, our study included a greater number of patients and a larger variety of systemic associations, as well as more comprehensive examinations of each patient, than that in previous studies. We included 54 patients with non-Sjögren’s DED and 52 with SS-DED and reported results for the OSDI, Schirmer test, and other corneal findings. This allowed us to evaluate the difference in tear lactoferrin levels across a wider range of patients and establish a correlation between lactoferrin levels and clinical findings. The use of lactoferrin levels as a biomarker for differentiating between different types of DED has several potential clinical applications. For example, it could help physicians to more accurately diagnose and manage patients with refractory DED associated with systemic diseases, such as primary or secondary SS. It could also aid in the development of personalized treatment plans for patients with DED based on their underlying systemic condition.

In the present study, there was female dominance in all groups. The higher female percentage in non-Sjögren’s DED and SS-DED were in accordance with previous findings that DED and SS are both more prevalent in the female population^36,37^. To avoid bias, the sex distribution in our control group was designed to be compatible with that of the other study groups. All DED subgroups in our study demonstrated lower lactoferrin levels compared to healthy controls, and lactoferrin levels in patients with SS-DED were even lower than those in patients with non-Sjögren’s DED. Because lactoferrin is secreted from main lacrimal, accessory lacrimal and meibomian glands, damage to these glands caused by acinar atrophy, tissue fibrosis and the infiltration of interlobular inflammatory cells in patients with SS can result in a decrease in tear lactoferrin levels^38^. Tear lactoferrin is mainly secreted from lacrimal glands, and the production decrease when the function and size of main lacrimal glands decline in patients with SS-DED^39^. In addition, patients with long-term SS demonstrate symptoms of chronic meibomitis or meibomian gland damage^40^. Severe meibomian gland inflammation may also lead to a reduction in tear lactoferrin. In contrast, in patients with non-Sjögren’s DED, there is usually no excessive or pathogenic fibrosis in the lacrimal glands^41^. These findings may account for the difference in tear lactoferrin levels detected in the present study and support the use of tear lactoferrin as a biomarker to differentiate between DED subgroups.

This study had a few limitations. First, SS-DED included cases of primary and secondary SS, and we did not further differentiate between primary and secondary SS. Second, although the impact of atrophy of the lacrimal glands in patients with SS-DED is known^10,36^, histological evidence was lacking in the present study. Further studies with lacrimal scintigraphy may help confirm our hypothesis of reduced tear lactoferrin levels in SS-DED. Third, this study did not exclude patients using immunosuppressive agents. Although our sensitivity analysis supports the robustness of our conclusion regardless of the use of immunosuppressants, further studies are required to better understand the effect of immunosuppressants on tear lactoferrin levels. Fourth, while the two DED groups had comparable mean age, the control group was not age-matched to the DED groups. Therefore, the potential impact of age on tear volume and lactoferrin levels may need to be taken into consideration interpreting the results in the control group. Finally, there is currently no perfect method for tear collection, and the collection method may vary among studies, thus direct comparisons of our results with that of other studies using different tear collection methods might not be feasible. Despite these limitations, we provide valuable information for point-of-care dry eye evaluations that can help establish a treatment protocol. In this study, we developed a cost-effective and highly sensitive method to evaluate tear lactoferrin levels and demonstrated its importance as a biomarker for differentiating patients with SS-DED from those with non-Sjögren’s DED by analyzing data from a large number of patients. In a future study, we hope to improve the sensitivity of our tool and collect a greater number of patient samples with a broader scope of AI diseases.

## Conclusions

Our newly developed photo-detection device is suitable for detecting tear fluid lactoferrin levels from samples collected through routine clinical practice and provides accurate measurements of tear lactoferrin, even in the lower concentration range, and could be used to differentiate patients with SS-DED from those with non-Sjögren’s DED, aiding in prompt treatment. Further studies with larger numbers of patients, longitudinal evaluations, and patients with different types of associated AI diseases are needed to help validate our findings and investigate the potential clinical application of this device.

### Supplementary Information

Supplementary Figures.

## Data Availability

Please contact Chao-Min Cheng (chaomin@mx.nthu.edu.tw) for requesting the data of this study.

## References

[1] Clayton JA (2018). Dry eye. N. Engl. J. Med..

[2] Craig JP (2017). TFOS DEWS II definition and classification report. Ocul. Surf..

[3] Benítez-Del-Castillo J (2017). Visual acuity and quality of life in dry eye disease: Proceedings of the OCEAN group meeting. Ocul. Surf..

[4] Nguyen AT, Elia M, Materin MA, Sznol M, Chow J (2016). Cyclosporine for dry eye associated with nivolumab: A case progressing to corneal perforation. Cornea.

[5] Wang L (2020). Autologous serum eye drops versus artificial tear drops for dry eye disease: A systematic review and meta-analysis of randomized controlled trials. Ophthalmic Res..

[6] Drew VJ, Tseng CL, Seghatchian J, Burnouf T (2018). Reflections on dry eye syndrome treatment: Therapeutic role of blood products. Front. Med. (Lausanne).

[7] Rong B (2018). Intense pulsed light applied directly on eyelids combined with Meibomian gland expression to treat Meibomian gland dysfunction. Photomed. Laser Surg..

[8] Lane SS (2012). A new system, the LipiFlow, for the treatment of Meibomian gland dysfunction. Cornea.

[9] Maskin SL (2010). Intraductal Meibomian gland probing relieves symptoms of obstructive Meibomian gland dysfunction. Cornea.

[10] Nguyen CQ, Peck AB (2009). Unraveling the pathophysiology of Sjogren syndrome-associated dry eye disease. Ocul. Surf..

[11] Wang L, Xie Y, Deng Y (2021). Prevalence of dry eye in patients with systemic lupus erythematosus: A meta-analysis. BMJ Open.

[12] Giansanti F, Panella G, Leboffe L, Antonini G (2016). Lactoferrin from milk: Nutraceutical and pharmacological properties. Pharmaceuticals Basel.

[13] Fillebeen C (1998). Lactoferrin is synthesized by mouse brain tissue and its expression is enhanced after MPTP treatment. Adv. Exp. Med. Biol..

[14] Teng CT (1989). Lactotransferrin gene expression in the mouse uterus and mammary gland. Endocrinology..

[15] Narayanan S, Redfern RL, Miller WL, Nichols KK, McDermott AM (2013). Dry eye disease and microbial keratitis: Is there a connection?. Ocul. Surf..

[16] Abrink M, Larsson E, Gobl A, Hellman L (2000). Expression of lactoferrin in the kidney: Implications for innate immunity and iron metabolism. Kidney Int..

[17] Vagge A (2020). Therapeutic effects of lactoferrin in ocular diseases: From dry eye disease to infections. Int. J. Mol. Sci..

[18] Flanagan JL, Willcox MD (2009). Role of lactoferrin in the tear film. Biochimie.

[19] Gillette TE, Allansmith MR (1980). Lactoferrin in human ocular tissues. Am. J. Ophthalmol..

[20] Kijlstra A, Jeurissen SH, Koning KM (1983). Lactoferrin levels in normal human tears. Br. J. Ophthalmol..

[21] Jensen OL, Gluud BS, Birgens HS (1986). The concentration of lactoferrin in tears of normals and of diabetics. Acta Ophthalmol..

[22] McGill JI, Liakos GM, Goulding N, Seal DV (1984). Normal tear protein profiles and age-related changes. Br. J. Ophthalmol..

[23] Versura P, Bavelloni A, Grillini M, Fresina M, Campos EC (2013). Diagnostic performance of a tear protein panel in early dry eye. Mol. Vis..

[24] Careba I (2015). Correlations between eyelid tumors and tear lipocalin, lysozyme and lactoferrin concentrations in postmenopausal women. J. Med. Life.

[25] Abe T, Nakajima A, Matsunaga M, Sakuragi S, Komatsu M (1999). Decreased tear lactoferrin concentration in patients with chronic hepatitis C. Br. J. Ophthalmol..

[26] Lema I, Brea D, Rodríguez-González R, Díez-Feijoo E, Sobrino T (2010). Proteomic analysis of the tear film in patients with keratoconus. Mol. Vis..

[27] Danjo Y, Lee M, Horimoto K, Hamano T (1994). Ocular surface damage and tear lactoferrin in dry eye syndrome. Acta Ophthalmol..

[28] Sonobe H (2019). A novel and innovative paper-based analytical device for assessing tear lactoferrin of dry eye patients. Ocul. Surf..

[29] Kijlstra A, Kuizenga A, van der Velde M, van Haeringen NJ (1989). Gel electrophoresis of human tears reveals various forms of tear lactoferrin. Curr. Eye Res..

[30] Sitaramamma T, Shivaji S, Rao GN (1998). HPLC analysis of closed, open, and reflex eye tear proteins. Indian J. Ophthalmol..

[31] Masoudi S, Zhong L, Raftery MJ, Stapleton FJ, Willcox MD (2014). Method development for quantification of five tear proteins using selected reaction monitoring (SRM) mass spectrometry. Investig. Ophthalmol. Vis. Sci..

[32] Yolton DP, Mende S, Harper A, Softing A (1991). Association of dry eye signs and symptoms with tear lactoferrin concentration. J. Am. Optom. Assoc..

[33] Yamada K, Takaki S, Komuro N, Suzuki K, Citterio D (2014). An antibody-free microfluidic paper-based analytical device for the determination of tear fluid lactoferrin by fluorescence sensitization of Tb3+. Analyst.

[34] Shimazaki J (2018). Definition and diagnostic criteria of dry eye disease: Historical overview and future directions. Investig. Ophthalmol. Vis. Sci..

[35] Mackor AJ, van Bijsterveld OP (1988). Tear function parameters in keratoconjunctivitis sicca with and without the association of Sjögren’s syndrome. Ophthalmologica.

[36] Smith JA (2007). The epidemiology of dry eye disease. Acta Ophthalmol. Scand..

[37] Jonsson R, Gordon TP, Konttinen YT (2003). Recent advances in understanding molecular mechanisms in the pathogenesis and antibody profile of Sjögren’s syndrome. Curr. Rheumatol. Rep..

[38] Sato EA (2010). Lacrimal gland in Sjogren’s syndrome. Ophthalmology.

[39] Delaleu N (2005). Sjogren’s syndrome. Eur. J. Oral Sci..

[40] Wang Y (2019). Clinical analysis: Aqueous-deficient and Meibomian gland dysfunction in with primary Sjogren’s syndrome. Front. Med. (Lausanne).

[41] Rocha EM, Alves M, Rios JD, Dartt DA (2008). The aging lacrimal gland: Changes in structure and function. Ocul. Surf..

