# Supplementary material for: Fluorescence-based reagent and spectrum-based optical reader for lactoferrin detection in tears: differentiating Sjögren’s syndrome from non-Sjögren’s dry eye syndrome
Source: Sci Rep. 2024 Jun 24;14:14505. doi: 10.1038/s41598-024-65487-2 (PMC11196714; doi:10.1038/s41598-024-65487-2)
Supplement: Supplementary file 1 — Supplementary Figures. [file 41598_2024_65487_MOESM1_ESM.docx]

**Supplementary material**


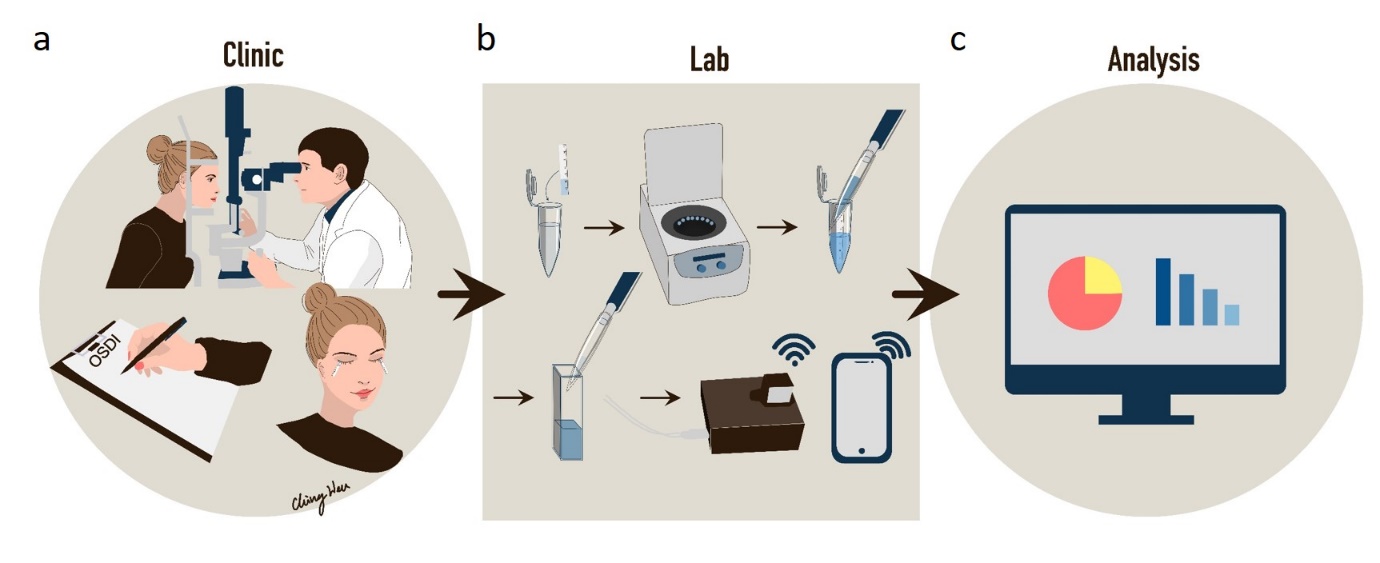


**Supplementary Figure S1. The study process flowchart of patient collection, sample detection and data analysis is depicted.** (a) Patients complete a slit lamp examination, Schirmer test, and OSDI questionnaire at clinics. (b) Tears retrieved from the Schirmer paper are resolved in 50 µL of dis-water for 15 minutes, centrifuged, and tested with the photo-detective device after being mixed with a TbCl_3_ solution in the laboratory. The fluorescence signal is detected and recorded on the APP. (c) Data are analyzed by one-way analyses of variance using Prism software (ver. 6.04 for Mac; GraphPad Software, Inc., San Diego, CA, USA). APP, Application; TbCl_3_, Terbium(III) chloride; OSDI, Ocular Surface Disease Index

**
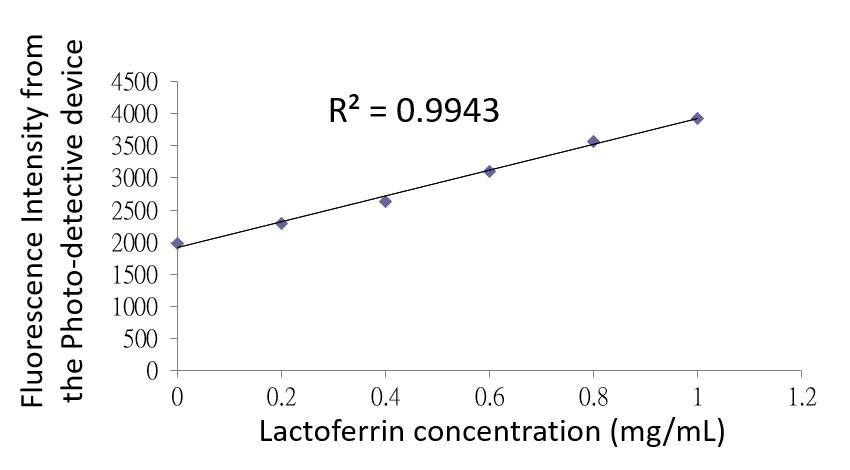
**

**Supplementary Figure S2.** **The calibration line of the photo-detection device is shown.**

Linear regression for the fluorescence intensity value of lactoferrin at concentrations of 0, 0.2, 0.4, 0.6, 0.8, and 1.0 mg/mL indicates the calibration line of the photo-detection device. R^2^=0.9943. The fluorescence intensity from the photo-detection device of the blank well is 1915.29. X-axis: concentration of lactoferrin samples (mg/mL); Y-axis: test value from the photo-detection device. ELISA, enzyme-linked immunosorbent assay
